# Music, imagery, and infertility: a qualitative inquiry into symptoms, treatments, and expressive therapies with infertility clinicians

**DOI:** 10.3389/fpsyg.2026.1778519

**Published:** 2026-06-23

**Authors:** Johanna Shriver-Halligan

**Affiliations:** Department of Expressive Therapies, Lesley University, Cambridge, MA, United States

**Keywords:** arts-based research, counseling, infertility, music, imagery

## Abstract

**Introduction:**

Infertility is a growing global issue that causes significant mental health distress. Individuals with infertility can experience stress, anxiety, depression, and post-traumatic stress disorder. Infertility counseling is a growing field, and the introduction of expressive arts therapies has allowed for a more in-depth understanding of the client’s infertility experience. Currently there is no research on the use of music and imagery with the infertility population, and very little research into the use of expressive arts with the same population. The aim of this study was to engage infertility clinicians through interview to explore current symptoms and treatments in the infertility community, and to explore how music and imagery as an arts-based method shaped clinician reflection.

**Methods:**

This qualitative phenomenological study used arts-based research methods to introduce the use of music and imagery with the infertility population, starting with clinicians currently working in the infertility field. Purposive sampling was used to recruit five infertility clinicians, four women and one man. Data were collected via a Zoom consisting of an artistic music and imagery experience and interviews. Using thematic *In Vivo* and photo-elicitation methods to analyze data, four clinical themes and three artistic themes were identified.

**Results:**

A total of five themes were identified, with subthemes- (1) The Complicated and Widespread Experience of Loss (emotional loss, loss of confidence, loss of fertility education, loss of genetic children because of my body, loss of social comfort, complicated structures of loss, colors/imagery representing loss), (2) Inconsistent Support Around the Infertility Experience (individual vs. couple/group, male vs. female, third party, clinical/medical, financial, isolating imagery), (3) The Long-term Mental Health Impact of Infertility (mental health, family), (4) The Long-term Mental Health Impact of Infertility (mental health, family, resolution in imagery), and (5) Reflection of Client Experience (facilitation of reflection through music, possible uses with clients).

**Discussion:**

Findings from this study demonstrated the potential of music and imagery as a positive tool for infertility mental health. Symptoms reported by clinicians corroborate past research on the impact of infertility on mental health, and participants identified a sense of loss in all areas of life. Participants discussed the music and imagery experience, with all noting the potential positives for use with clients. Limitations to this study and recommendations for future research were identified.

## 1 Introduction

Infertility, defined as the inability to achieve a pregnancy following 12 months of unprotected sex (World Health Organization, 2023), is a uniquely medical and social experience leading to significant social stigma and shame (Yokota et al., 2022). High levels of stress, depression, and anxiety within the infertility community are comparable to stress reported by those with heart disease, cancer, and human immunodeficiency virus diagnosis (Holley et al., 2015). There is also a high risk for posttraumatic stress disorder (PTSD) following pregnancy loss during infertility treatment (Öztürk et al., 2021). Social and emotional stress from infertility may be compounded by losses like miscarriage or personal identity disruption from the loss of the family dream (McBain and Reeves, 2019; Öztürk et al., 2021; Wallis, 2021).

Presently the use of expressive therapies, and music in particular, as an arts-based platform, has not been explored within the infertility community. However, studies have shown that listening to music can reduce stress and anxiety and elicit involuntary imagery (not defined as positive or negative) (Fallon et al., 2020; Filippidi and Timmers, 2017; Matoso et al., 2022), and that Translating mental imagery into a physical drawing while listening to music has been shown to increase situational understanding and greater focus on assigned tasks, such as learning a new skill or memorizing facts (Han, 2016).

Over the years, clinicians have identified that a multiple treatment model is best when treating the mental health side of infertility, frequently combining mindfulness and traditional verbal psychotherapy methods (Dube et al., 2021; Kalhori et al., 2020). Limited studies have examined expressive therapies as a treatment for modality for infertility distress (ID) (Hughes and Da Silva, 2011; Streeter and Deaver, 2018; Terzioğlu and Özkan, 2018), and expressive therapy techniques, when used alongside more traditional therapy methods, can enhance these treatments (Dube et al., 2021). The aim of this study was to engage infertility clinicians through interview to explore current symptoms and treatments in the infertility community, and to explore how music and imagery as an arts-based method shaped clinician reflection.

### 1.1 Infertility counseling

The desire for comprehensive mental health treatment to ease the distress of infertility has long been requested by patients, with some countries forming professional associations in the 1990s (Thorn, 2009). Most of the research and treatment for infertility focuses mainly on the medical aspect, historically referred to as the medicalization of the infertility condition, especially in women (Parry, 2004). Current approaches to treatment for ID utilize a variety of counseling methods, such as cognitive-behavioral therapy (CBT) and mindfulness-based therapy (MBT), with a combination of these identified as the most effective treatment currently (Wallis, 2021).

For individuals and couples, counseling can help reduce symptoms of ID. The strain of infertility on individual functioning can also lead to stress in couples, causing discord, sexual dysfunction, and arguments about how to proceed with treatment (Anguzu et al., 2020; Casu et al., 2019; Dawson et al., 2005; Koert et al., 2019; Koser, 2020; Shapiro, 2009). It has been historically noted that traditional counseling methods, such as CBT, have not always been effective with the infertility population due to the extreme variations in the infertility experience and the feedback from clients that more traditional approaches felt invalidating of their experience (Boivin et al., 1999; Burnett, 2009; Dube et al., 2021; Gibson and Myers, 2000). In response, research has started to explore an eclectic mix of therapeutic methods to identify more effective treatments for ID (Golshani et al., 2021; Mosalanejad et al., 2012; Streeter and Deaver, 2018).

While there is discussion of the importance of treatment and of different outcomes with different therapy methods, few articles assess the experience and approach to treating ID from the clinicians who provide treatment. Studies have explored the reasons individuals experiencing ID may not have sought out counseling while simultaneously identifying parts of managing ID that could benefit from counseling, such as locus of control and proactively preparing for the emotional side of infertility (Born et al., 2018). A study conducted by Dube et al. (2021) noted it was one of the first to include clinicians and treatment methods in the interview process, providing a direct look at the techniques incorporated into ID treatment and the reasoning behind them. A goal of this study was to engage with clinicians treating individuals with ID to understand how they approach counseling this population, and what methods have been effective with their clientele.

### 1.2 Current treatments in infertility counseling

While CBT and MBT are the most common treatments for ID, several other treatments have been studied and have shown promise. Social support emerged as an essential part of coping with ID, with individuals with infertility identifying helpful and non-helpful social behaviors (Abraham, 2019; Borowczak and Rotoli, 2022), including seeking community through social media sites like Instagram (Perone et al., 2021).

Emotion-focused therapy was conducted with six couples in Iran and examined the sharing of emotions and restructuring of emotional bonds needed between partners. A comparison of Depression Anxiety Stress Scales (DASS) scores between the groups showed significant changes in the intervention (Soltani et al., 2013). Another study using information about infertility treatment to reduce anxiety showed significant decreases in anxiety directly following treatment and 2 weeks after (Hamdieh et al., 2009). The introduction of grief training is another approach that is being added more frequently to infertility counseling, as men and women may grieve the same but present differently in public (James and Singh, 2018; Tian and Solomon, 2020). Infertility may cause loss in a variety of ways, and a study on disenfranchised grief noted that women with infertility often feel invisible, and their grief is not legitimized or seen by the American public at large (McBain and Reeves, 2019).

### 1.3 Arts-based research and emotions

Arts-based research (ABR) and methods is an umbrella term where a creative worldview forms the basis of inquiry, and the artistic process shapes the research process. A cornerstone of ABR is the generative and emergent process, which allows for unexpected and surprising methods and results (Leavy, 2017; Viega and Forinash, 2016). Studies using art-based and expressive methods have demonstrated that art has the potential to be an effective way to gain an understanding of a lived experience in a meaningful way (Carew, 2018; Estrella and Forinash, 2007), and can be used within the wider scope of qualitative research to aide in creating rich results and approaching issues in research, especially those with stigma or intense emotional involvement. Through multiple angle approaches, researchers can use a creative process platform to explore different individual levels of feeling safe when applied to human emotional states, such as depression (Dieterich-Hartwell, 2023; Viega and Forinash, 2016). Furthermore, emotional regulation and engagement occur in emotional situations, and infertility is a highly emotional situation that impacts both clinicians and clients (Fancourt et al., 2020). Using artistic engagement in conjunction with clinicians may identify areas of emotional regulation with clients and clinician perceptions of client experiences. Fancourt et al. (2020) found that women, younger people, and those in lower socioeconomic brackets engage more frequently in creative activities, such as accessing music and art. This group is a critical component of the infertility population, and introducing creative engagement to clinicians may allow for more effective use of creative arts to manage the emotions of the infertility experience.

In a personal study exploring artistic inquiry and depression, it was noted that artistic inquiry is essential to making sense of human conditions (Dieterich-Hartwell, 2023). Artistic space was identified as fluid, allowing for perspective shifts differing from primarily verbal techniques.

Identifying the impact of artistic engagement on clinicians, researchers addressed many benefits, including engaging with self, working through countertransference, feeling restricted, creating safe communication pathways with clients, and allowing for a new understanding of clients’ situations or conditions (Dieterich-Hartwell, 2023). Countertransference especially may be shared among infertility clinicians who may have experienced personal infertility alongside their work. Dieterich-Hartwell (2023) frequently stated that artistic engagement is accessible and essential to developing effective treatments for infertility distress with a broader reach. To gain understanding of the impact an arts-based approach could have on the emotional state, clinicians were chosen as the target audience for this study to gain their feedback prior to attempting a music and imagery experience with individuals experiencing ID. As noted above, Fancourt et al. (2020) identified noted the high emotional impact of infertility on both clinicians and clients, indicating artistic and creative activities may assist in regulating emotions for all members of the treatment dyad.

### 1.4 Music therapy and music and imagery in reproductive research

While current music therapy research has not studied reproductive mental health in depth, there are studies that have looked at the use of music to address parallel symptoms. In a study on the relationship between music and emotions, Lavy (2001) framed music as human engagement with the environment within a web of thoughts and knowledge. Lavy noted that music is perceived as a narrative and that emotional shifts can be detected as in human speech. A study assessing the effectiveness of organizational music therapy for nurses in a public hospital, in which 71.6% showed stress levels in the resistance stage based on Lipp’s Stress Symptoms Inventory for Adults (LSSI), was conducted (Matoso et al., 2022). Following receptive music listening in the hospital setting, stress levels were re-evaluated at 30 days post-intervention using the LSSI, and significant decreases in stress were noted.

Listening to music was found to decrease stress levels following a stressful task better than music improvisation or no intervention, regardless of the style of music played (Diaz, 2015; Fallon et al., 2020). These findings align with previous studies: music listening consistently reduces stress (Küssner and Eerola, 2019; Matoso et al., 2022; Vamvakaris, 2020; West, 2003), suggesting it may facilitate more open discussion and safer management of the stressful topic of infertility. Infertility is an emotional experience and may inspire strong emotions in both clients and counselors, especially if counselors have also experienced infertility. Pairing music with a reflection-based task may allow for a more accurate, engaged, and honest assessment of current issues and treatments for ID.

Examining the role of imagery alongside music and music listening, Filippidi and Timmers (2017) identified a connection between music and involuntary imagery, finding that music listening often leads to involuntary imagery shaped by one’s listening experience. In a study with classical performers, imagery facilitated a more streamlined cognitive process and enhanced positive learning (Davidson-Kelly et al., 2015). In a second study looking at the cognitive process while engaged in music and imagery, students completed an assignment drawing out images that came to mind while listening to music. Results indicated students were able to express their experience with greater accuracy and understanding with the use of music and imagery, vs without music and drawing (Han, 2016). As music often evokes involuntary imagery (Filippidi and Timmers, 2017), pairing music listening with visual imagery as a research method may allow participants to connect with the cognitive and emotional aspects of infertility, creating a deeper, more holistic view of the phenomenon being explored.

Within the scope of music therapy research, studies using both the Bonney Method Guided Imagery and Music (BMGIM) and the Continuum Model of Guided Imagery and Music (CMGIM) have been conducted. Helen Bonney developed her method in the 1970’s, while CMGIM was developed as an adaption of BMGIM by Frances Goldberg and Dr. Lisa Summer, both students of Helen Bonney, in the 1980’s (Montgomery, 2012; Summer, 2010; Beebe and Wyatt, 2009; Howard, 2025). The BMGIM uses a program of music to create a series of images, with clients moving into an altered relaxed state. In CMGIM, guided music and imagery can be used alongside music and imagery, allowing for the therapist to tailor the experience to the needs of the client (Summer, 2015). Using a repetitive form of music, one image is created that focuses on a supportive, re-educative or reconstructive work of the client (Howard, 2025).

A systemic review completed by McKinney and Honig (2017) identified nine studies that demonstrated positive outcomes using BMGIM to manage anxiety, depression, sense of coherence, and interpersonal problems. Studies completed by Beck et al., 2018a,b explored the use of BMGIM with work related stress and PTSD in the refugee community, with decreases in anxiety, depression, stress, and PTSD symptoms showing a decrease from pre intervention assessments in post testing. A case study using CMGIM to address issues of anxiety and self-doubt showed the use of CMGIM to explore and integrate these feelings, decreasing the overall effect of anxiety and stress and elevating positive self-resources (Howard, 2025). A series of historical and current studies have explored the use of music and imagery to process motherhood, postpartum, and pregnancy: all issues that are impacted by infertility, prior loss, or other reproductive trauma (Short et al., 2025; Short, 2023).

Because there is minimal research on the use of either music therapy or guided imagery protocols specifically with the infertility population, this pilot study sought to begin to address this gap through introducing music and imagery in hopes of developing a more specific guided imagery protocol. While there are no specific GIM studies in this area, multiple studies using music and imagery methods have addressed symptoms of anxiety, depression, grief, and PTSD: all symptoms identified and experienced by the infertility population. Working to develop a more clinical use of music and imagery, this exploratory study looked to introduce the use of music and imagery with clinicians rather than the clinical population. This will enable clinicians to see if this method is suitable for the infertility community.

## 2 Materials and methods

This research explored the use of music and imagery the use of music and imagery to express the infertility experience for these participants, using data on current infertility distress (ID) symptoms and treatment approaches. A phenomenological qualitative method was used, consisting of a music-and-imaging experience and an interview. The research questions explored were (1) What are the experiences of infertility clinicians reflecting on their clients’ infertility experiences, and (2) How did the use of artistic engagement shape the reflection process?

The use of a qualitative phenomenological approach was chosen to put the emphasis on the lived human experience of infertility (Alase, 2017; Blumenfeld-Jones, 2016; Harasym et al., 2024). This study employed a hermeneutical phenomenological approach, with included arts-based research (ABR) methods to enhance the traditional approach of qualitative methods and assist in eliciting participant responses in interview (Harasym et al., 2024). The use of arts-based research also allowed for data that was multilayered and rich, creating a methodological approach that included multiple angles. The use of ABR within the qualitative design provides built in reflexivity for the researcher, as they must examine their own reactions to the art generated (Blumenfeld-Jones, 2016; Harasym et al., 2024). For this study, the researcher completed a music and imagery exercise following the completion of every interview, allowing their reactions and feelings to the interview to be contained in the process and out of the analysis. While this study did not use a BMGIM or CMGIM protocol or engage in music therapy, it is the hope of the researcher this study may provide a baseline in developing a future protocol incorporating related music and imagery elements by starting to create an understanding of how music and imagery is experienced by the infertility community.

### 2.1 Participants

Five participants were interviewed for this study, using pseudonyms to maintain confidentiality (Table 1). All were licensed clinicians working with clients with infertility. All participants identified as White, four identified as female, and one identified as male. Participants were recruited from across the United States and worked in various settings, including private practice, therapeutic groups, and referrals from clinics. Two participants were expressive arts therapists, two were social workers, and one was a licensed mental health counselor. Three participants had a history of personal infertility, two did not.

**Table 1:** Participants.

Name	Licensure	Region	Expressive therapist	Setting	Personal infertility
Liliana	LMHC AT- R	South	Yes	Private practice	No
Eloise	LMHC REAT	New England	Yes	Private practice, groups	Yes
Matteo	LCSW	New England	No	Private practice	No
Maddi Lynn	LCSW	Pacific Northwest	No	Clinics	Yes
Juliette	LMHC	Mountain	No	Clinic referral, private Practice	Yes

### 2.2 Procedure

The research questions, music and imagery experience, and interview questions were developed by the researcher and approved by the Lesley University Institutional Review Board (IRB). Following IRB approval, clinicians specializing in infertility treatment were recruited via email. Once a clinician expressed interest in research participation, informed consent was sent, and upon return, an interview was scheduled via Zoom. Participants also received oil pastels and a sketchbook via mail, which they kept after the interview. Oil pastels were chosen for the bold color and ease of use. To start the interview, participants were reminded that it was being taped, given the opportunity to ask questions, and revoke consent if desired. Following in-person assent, a 60–90-min interview and music and imagery experience were conducted.

At the start of the music-and-imagery experience, the researcher introduced the musical selection and explained the process. A prompt was given by the researcher, followed by the musical selection played three to four times in a row with no pause (Supplementary Appendix 1). The participant drew while the music played, with the researcher silently observing. The musical piece chosen used an ABA composition format, featured one instrument, and stayed within major chord structures. The musical structure and instrumentation provided stability for the listener and supported the reflective nature of the imagery prompt. The music and imagery exercise was completed at the start of the interview to allow an authentic reaction to the music and imagery experience before through the interview process. Arts-based research methods such as music and imagery can be helpful in facilitating research on topics that can be very personal or stigmatizing, such as infertility (Carew, 2018; Estrella and Forinash, 2007; Fancourt et al., 2020; Viega and Forinash, 2016).

A semi-structured interview was conducted following the artistic experience, using interview questions to guide the conversation (Supplementary Appendix 2). Use of the semi-structured interview is an important tool in qualitative research, allowing for a focused interview that still gives space to explore ideas or thoughts participants or researchers may come across as they talk (Adeoye-Olatunde and Olenik, 2021). Participants were asked about their practice, the type of clients they see, and the treatment techniques they use. Participants were asked about common symptoms they see between between clients, and if they had a personal history of infertility. They were also asked a variety of questions about the music and imagery experience, including the role of music, the process of reflection through art, and whether they felt this method would be helpful for individuals experiencing infertility. Participants were asked the standard demographic questions and given the chance to share information or thoughts they had not previously shared.

### 2.3 Data collection

Data collection was focused on two separate areas: (1) Interview, and (2) Imagery. Data collected from the interview questions provided clinician reflection through a non-arts-based method. In contrast, imagery coding was derived from themes in the artwork as observed by the researcher, the participant’s analysis of their image, the colors used in the image, and participant descriptions of their image. Data was collected in clinical and imagery categories to answer both research questions effectively: what clinicians are currently experiencing clinically and the impact of the artistic engagement through the images created. The images created were an additional layer of data that offered more insight into the lived experience of infertility. While many of the participants had experienced infertility themselves, they also spent time and space in the experiences of others, allowing for unique insight on the topic. The researcher chose to complete the artistic engagement prior to the interview to decrease the nervousness of the participant around art, and to allow the image to manifest naturally, prior to an in-depth conversation on infertility experiences.

### 2.4 Data analysis

The principal researcher collected and analyzed all data manually over 6 months. A thematic analysis using the *In Vivo* method (Saldana, 2021) was the primary analysis method for the interview questions. Photo-elicitation (Glaw et al., 2017) was used alongside *In Vivo* from the participants regarding their image, through the use of the image to guide the conversation around the infertility experience. The researcher read each interview twice, with the first read focusing on keywords and phrases that were repeated frequently or emphasized by the participant. The second read concentrated on the highlighted phrases from the first read and grouped them into themes. Data were coded on a rolling basis following interview completion, and subsequent interviews were added as they occurred. Themes were tentatively formed as data were coded, and phrases from the interview were sorted into initial themes based on similar statements. As larger themes emerged, subthemes broke out based on participants’ more specific experiences under each umbrella theme. Following the completion of artistic analysis, artistic data were blended into the larger themes identified. The researcher continued to group statements and reduce similar data points until arriving at five themes with sub-themes.

*In Vivo* (Saldana, 2021) was chosen as the primary clinical analysis to further highlight the researcher goal of framing the experience through the lens of the participant. Results were formed from the statements of the participants, allowing for their experience to guide the analysis and further reduce researcher bias on data (Blumenfeld-Jones, 2016; Harasym et al., 2024). Arts-based research methods can be easily analyzed alongside and within the qualitative method, and photo-elicitation was used as a visual analysis method alongside *In Vivo* to highlight the participant descriptions of their image and how they created it (Alase, 2017; Blumenfeld-Jones, 2016; Glaw et al., 2017; Harasym et al., 2024). From the researcher perspective, the repeated use of symbols and colors across the images was analyzed, alongside the participant explanations around the symbols and colors they chose, and the reasoning behind them.

At the conclusion of the analysis period, the researcher reviewed data to ensure it had reached saturation and identify the areas of credibility and transferability. The data collection period lasted 6 months, allowing for extended time in the field interviewing and the collection of data with thick description. Triangulation was achieved using music and imagery to collect data from an additional point of view alongside verbal data, and the participation of infertility counselors from a variety of counseling perspectives. The inclusion of participants who worked in private practice, clinic embedment, and group therapy allowed for information from a variety of client experiences, and the participation of clinicians with differing clinical approaches offered thick description on the experiences processed through both expressive and traditional clinical approaches. Additionally, the participants covered a wide variety of clinical cases with both male and female patients, an inclusion not seen frequently in past studies (Garcia, 2021; Holley et al., 2015; Harlow et al., 2020; Bell, 2020, Lucia and David, 2022).

### 2.5 Researcher positionality

To frame this research through the lens of the researcher, the following positionality statement is offered. As a heterosexual White woman with a personal history of infertility and recurrent pregnancy loss, this research carries a personal feel. My personal experience of reproductive trauma and the use of music and imagery to process is how this paper came to be, and as such, I have aimed to treat this important topic and community with respect and empathy. Infertility comes from a variety of experiences and emotions, and many of those experiences are underserved and under researched currently. The experience of the White heterosexual woman or couple is the predominant account shared, and this account does not adequately express or explore the impact race, socioeconomics, martial/relationship status, culture, or sexual and gender identity have on the infertility experience. Each of these factors can and does significantly impact how infertility is experienced and supported socially. It is my goal as a researcher to bring awareness and understanding to the experience of the global infertility community with respect and ethical research. Emotions are layered and complex in infertility, and to protect the participants and community, this research was conducted under the purview of the Lesley University Institutional Review Board, as well as the oversight of my dissertation committee. As a member of the infertility community, reducing personal bias through the design and completion of this project was a critical component. I completed a personal music and imagery experience following each interview, to create a container for any emotions stirred by the content. To allow for a clear perspective while analyzing the data, I chose to wait at least 1 week before starting to analyze either the interviews or images. Through monthly meetings with the chair of my committee, I worked to identify further personal bias that might be unclear to me and took breaks from both data collection and analysis when needed.

## 3 Results

A total of five themes with subthemes were identified (Table 2). The five themes are as follows: (1) The Complicated and Widespread Experience of Infertility Loss (emotional loss, loss of confidence, loss of fertility education, loss of genetic children because of my body, loss of social comfort, complicated structures of loss, loss in imagery), (2) Inconsistent Support around the Infertility Experience (individual vs. couple/group, male vs. female, third party, clinical/medical, financial, representation in imagery), (3) The Long-term Impact of Infertility (mental health, family, resolution in imagery), (4) Personal Impact of Infertility on the Clinician Experience (personal reproductive story, treatment approach, sense of self in imagery), and (5) Reflection on the Client Experience through Music and Imagery (facilitation of reflection through music, possible uses with clients). Artistic data analyzed from the images represented many of these same themes, using color and symbolism. Many of the colors and symbols used in the images had multiple meanings and fit into multiple themes.

**Table 2:** Results.

Theme	Sub-theme
The Complicated and Widespread Experience of Loss	(1) Emotional loss (2) Loss of confidence (3) Loss of fertility education (4) Loss of genetic children because of my body (5) Loss of social comfort (6) Complicated structures (7) Loss in imagery
Inconsistent Support Around the Infertility Experience	(1) Male vs. female (2) Individual vs. couple/group (3) Clinic/medical (4) Financial
The Long-Term Impact of Infertility on Individual and Family Mental Health	(1) Mental health (2) Family
The personal Impact of Infertility on the Clinicians	(1) Personal reproductive story (2) Treatment approach(3) Sense of self in imagery
Reflection of Client Experience through Music and Imagery	(1) Facilitation of reflection through music (2) Possible uses with clients

### 3.1 The complicated and widespread experience of loss

Loss in this study included emotional and physical loss, loss of educational opportunities, the experience of shifting core beliefs about fertility, and was the largest theme represented both in interview and imagery. Often loss included feelings of sadness or grief but also was identified as the loss of an opportunity to gain new information for treatment or needed support. Loss was a theme identified by all five participants as a significant impact resulting from the infertility experience.

Emotional loss was identified by multiple participants and is defined in this study as the sadness, regret, and grief associated with loss of abstract concepts such as early pregnancy, missed life opportunities, and trust in the body. Juliette summarized emotional loss aptly in the following quote, noting the extensive scope of sadness and grief associated with emotional concepts:

The clients say I lost trust in my body. I lost a pregnancy. I lost the future vision…There is that emotional loss. I lost what I thought Christmas was going to look like this year, I lost that my sister- in-law and I were due 2 weeks apart. I lost…. There is so much loss. I say you have lost a vision for what your life was supposed to look like. There is that emotional loss of what does the future hold, and how do I navigate these next years and months with this unknown, so there is so much, so much loss involved, even if you have never been pregnant.

Other participants agreed, with Matteo noting, “It is just kind of like a no-hope kind of thing.” Maddi Lynn identified guilt as a central piece of emotional loss in infertility, saying, “I see a lot of guilt, like, I wish I hadn’t waited. I wish I hadn’t had my career first.”

Participants also discussed a loss of confidence in advocating, both in medical settings and within relationship dyads. This type of loss is defined in this study as feeling unable to speak up or advocate for needs in medical settings or relationships. This included sexual demands from partners and doctors disregarding patient feelings. Maddi Lynn and Juliette both shared their clients’ experiences with touch saturation, from partners and medical procedures, when the consequences make it hard to say no. Maddi Lynn shared, “I think we talk a lot about it (the feeling of being touched out),” and Juliette pointed out, “With the pokes and prods and the blue dye tests- there’s a legitimate fatigue to it, and it is hard to say no; I don’t want to be intimate with my partner.” Liliana noted she often has clients who struggle to feel confident in medical settings, after the clients having not been believed by previous doctors concerning their condition, saying, “I feel like I end up with all the clients who’ve been to like 47,000 providers who have told them that they’re full of shit.” Liliana further spoke about her clients experiencing medical post-traumatic stress syndrome due to “medical gaslighting clients losing confidence in medical settings, with clients experiencing “that pushback like, your doctor says that (procedure’s) useless, but then other doctors are selling it, and you’re like, what do I do?” Eloise shared a similar experience a client had, saying:

She (the patient) was talking about going to the doctor. The doctor basically assumed that they would go in one direction and just set up appointments for them moving in that one direction, (the doctor) not saying, let us put all the options on the table, and she felt like no, no, let us back up.

A lack of fertility education and of history from family and societal factors was another loss identified, with all participants noting that family histories are not shared with later generations, resulting in a lack of awareness of family fertility issues. Participants indicated that infertility carries a stigma, discouraging people from sharing fertility issues. Matteo commented,

I think that is maybe the hardest part…. There is this giant misunderstanding of how pregnancy works in our world. There is an inherent belief in our system and our society that if you meet someone you like, you get pregnant, and that is it. And that is not true; it is a false lie (the idea that pregnancy is easily achieved for everyone). And that is not told to them. Having kids is not as easy as our parents said.

He specified that family education is a missing piece with his clients, saying parents “don’t want to share (a bad fertility history) because they think it’s gonna hurt your feelings. So, you will never know they had a hard time, because they don’t believe in telling you about the hard times.” Maddi Lynn agreed, noting that, “I think there’s…a lot of people are getting better and better education, but a lot of women have been sold a bill of goods on you can have it all and you can wait, and you can’t.” Liliana noted her clients spoke about trying (to conceive), saying their mothers had an easier time, before realizing “this process is difficult.” Maddi Lynn and Eloise both spoke on how cultural norms can also impact the process of sharing fertility history; Eloise spoke about the impact of cultural understanding of fertility, saying,

People in their families, you know, did not know a lot about it (infertility) or came from other countries. They did not really have access to this care (so it is hard) to just explain that, you know, so I think they (the clients) might have a comfort level with it, but maybe their family does not so much, and how do you navigate that?

Participants all spoke at length about the loss of genetic children experienced by their clients, defined in this study as the loss of the expected function of the reproductive system to conceive and carry a pregnancy. Participants identified recurrent losses as part of this, with Juliette noting her clients experienced “a decent amount of pregnancy loss in that first trimester.” Eloise concurred, saying many of her clients experienced multiple pregnancy losses before successfully carrying a pregnancy to term. Eloise and Liliana both spoke of client frustration related to one’s body, with Liliana noting, “They have a lot of anger about their body malfunctioning, and Eloise noting that her clients “feel disconnected from their body or frustrated or angry that…. Their bodies are not doing what they wanted them to do.”

Matteo further noted that some of his clients decided against further genetic children after experiencing trauma, with one client successfully having a child, followed by a stillbirth. He tied this to the loss of more genetic children because of the loss of trust in the body. Maddi Lynn identified a unique experience she saw while working in clinics, where once a successful IVF cycle was completed, a client terminated the pregnancy because her body struggled with pregnancy. She talked about “how they’re mourning. They didn’t like being pregnant. They thought they wanted this and (in the end) they don’t.” Matteo noted there was some difference in how his male clients approached their bodies’ malfunction contributing to infertility compared to his female clients. One male client, “very matter of fact- if it (infertility treatments) doesn’t work, I won’t have more kids,” versus his female clients, who talked a lot about “frustration that their bodies aren’t working the way they are supposed to.”

A smaller subset of loss identified was the loss of social confidence and comfort, with both Eloise and Matteo stating their clients frequently discussed this. Eloise gave an example of a client who was pregnant at Christmas with her husband’s family, noting that if she just relaxed, she would get pregnant, and the client expressed hesitation to spend time with her in-laws following this interaction. Eloise said that socially known infertility leads to a lot of pressure, “Just like the pressures of Johnny says ‘don’t do that, don’t exercise,’ you know, just anything you do feels connected to it.” Matteo noted it can lead to clients avoiding family gatherings; “So if people are having babies and baby showers, come hang out with my newborn kind of thing, they (the clients) tend to shy away from that, because it’s uncomfortable.”

The final aspect of loss identified by the participants was a layered, complicated, all-encompassing loss. This form of loss was identified in this study as loss that touched all aspects of life, often overlapping with grief and support. Participants identified that loss often overlaps with other parts of the infertility experience, such as grief, support, and long-term impact on those experiencing infertility. When examining support and loss, Eloise spoke of the feeling of isolation when starting treatment. Eloise recalled that her clients’ experiences were like the one she experienced while going through infertility. She remembered “sitting in the waiting room, and everyone was just looking at the ground; I kept trying to make eye contact with somebody, anybody.” Juliette’s clients talked of wanting to feel hopeful, but “Just that idea of hope is scary, so how do you balance allowing for that hope or that excitement or that optimism?” Juliette often sees grief and loss together, telling her clients.

You’re not going to save yourself from grief if you do not get excited or do not get happy. That does not prevent you from grief. There is a grief from putting your life on hold, a grief in the I could have, would have, should have, done all these things.

Matteo and Maddi Lynn both observed that loss can lead to long-term mental health impacts, Matteo saying, “You can always get a new job, you can find a new apartment, not so with the fertility stuff.” Additionally, Madi Lynn found her clinical clients worried about falling behind (on the life timeline), especially after pregnancy loss. She stated, “A lot of my patients don’t even want to get pregnant, because they just assume they’re going to have a loss and then I won’t be able to get into the next IVF cycle.”

Loss was widely represented in the images produced, with all participants identifying colors or symbols of loss, sometimes both. Loss represented the largest space in the imagery produced, with loss sometimes overlaying varying emotions such as hope, demonstrating the complicated nature of loss in infertility. Emotions were identified by participants both as general emotions and emotions tied to specific parts of the infertility process. Blue, for example, felt “like the sadness (of infertility)” to Maddi Lynn, and Matteo said to him, “It felt like a puddle.” Liliana saw blue in her image, both in a lake and mixed with pink, calling it “little slivers of hope,” and Juliette saw the coolness of the blue as “a calming peace.” Eloise noted she had a mixture of all colors, but blue “felt like the grief, sadness, but also moments of connecting and not feeling alone.” Red was another color identified by many of the participants, generally associated with anger, a barrier, or depression. Liliana noted red birds in her image, “They were the anger, but I wish there were more room for the anger.” Maddi Lynn said the red to her felt “like how low can I go, how low will I allow myself, my family, or my marriage or whatever to go.”

In some images, colors associated with loss also had dual meanings such as Juliette, who saw red as both a hopeful thing, “as a therapist helping people to get to a place of peace,” and as “a barrier.” Liliana also talked about the red being “people’s disappointment in their bodies.” Maddi Lynn and Juliette both identified red as meaning blood, a period, or a miscarriage. Maddi Lynn talked about green as an envious feeling, “I drew different levels of green on green, green with envy. You’re envious that you want something, someone else, that you can’t have.” Juliette identified green as “a very thin line of hope,” and Eloise, Matteo, and Liliana all had green in their images, but did not assign an emotion to it. Other colors identified were pink, with Liliana noting she “felt it hopeful, a little bit.” Eloise said she could not separate the colors into different emotions, but “seeing them all together felt hopeful.” Maddi Lynn added orange and yellow, saying they represent “brightness and hope. Orange isn’t happy; it isn’t sad.” Black had the most consistent response across the images, representing a barrier causing frustration and loss. Gray was incorporated by Maddi Lynn as the embryos, noting, “I work in a clinic, and that area just feels gray.” Black was identified by all five participants as barriers, failed treatments, and grief/loss. Juliette noted in her image that the black lines “are, oh wait, are we going back now? It’s a block.” Liliana had mountains drawn in black, representing the “uphill struggle of my clients.”

### 3.2 Inconsistent support around the infertility experience

Support was a theme identified in various ways within the infertility experience by all five participants. Support is defined in this study as support from the family unit, financial support, and the types of support individuals experiencing infertility request. The largest major sub-theme within the support theme was female vs. male support concerning infertility, with women being the majority of individuals seeking professional support.

All five participants stated their female clients struggle with anxiety, depression, and trauma. Matteo and Liliana both noted that they try to help their clients understand that anxiety is normal, with Matteo saying, “You’re anxious for a reason; it is because you care.” Liliana noted a similar feeling in her interview, saying, “(I tell them) if you weren’t anxious and depressed about that (infertility), I feel like I would be more worried about you.” Maddi Lynn and Juliette also spoke of anxiety among their clients. Maddi Lynn saw many of her clients during active cycles, during their “wait” time between procedures. She said her goal for her female clients was to focus on living during the two-week wait, saying, “The two-week wait is horrible, we all know that, so we just try to get coping skills in place.”

Eloise identified her role in helping her clients understand that strong emotions are normal, and even in the case of the male partner being infertile, women struggle to manage their role in the process. She commented, “I’ve worked with women who have had to go this route (IVF) because their partner has a medical issue, and…. the feelings.” Liliana spoke about her female clients having a significant amount of anger, stating, “It is one of the biggest pieces in the timeline of (mental health) treatment.” Liliana also discussed another aspect of the treatment timeline- the physical stress on her female clients in addition to emotional stress. She noted, “The physical stress on the body for women…it is hard (like the mental toll).”

Matteo was the only participant working with male clients, noting that males tended in his practice to focus more on their partners’ issues and treatment than their own. “Men are more focused on the person (their partner), and when I ask how they are… we’re learning that men have evolved to have feelings.” Matteo wondered if it were easier for men to talk to other men about fertility issues: “It might feel, if a man is having fertility problems, it might feel emasculating to tell a woman that versus a man. There’s definitely a stigma there.” He further spoke to his experience of male clients being defensive, “Like yeah, I don’t know if it is a defense thing where like, they don’t want to admit it really, really affects them.”

Individual therapy and group/couple therapy were other supports identified. While individual support focused on processing specific emotions and situations, group counseling offered a different element of support. Eloise, Juliette, and Maddi Lynn all provided both group and individual counseling. All three agreed that group interaction was powerful, offering unity and support in the otherwise isolating issue of infertility. Group support “provides some comfort in knowing it is not just you, where everyone speaks the same language. It is a powerful thing” (Eloise). Juliette and Eloise both spoke to the benefit of group therapy beyond active treatment. Eloise has group counseling for clients who become pregnant, become mothers, and become parents after infertility loss and challenges. Juliette offers group sessions for postpartum and pregnancy loss, both in the hospital and her private practice.

Participants also examined support from medical and infertility clinics, with some responses positive and others negative. Participants defined positive and negative support as feelings of connection and warmth, respectively, interpreted as positive by clients, while support perceived as cold or unfeeling was described as negative. Some of Liliana’s clients identified multiple chronic conditions requiring support at odds with infertility treatment. “They have to get all these different procedures, and they have to tackle issues like tissue growing places (uterine lining attached to organs outside the uterus)” (Liliana). Maddi Lynn noted her clients may need medications to support mental health associated with infertility, and her role is to say, “I know you are so worried about meds, but there are other people who are on meds and pregnant.”

Juliette and Liliana both spoke about the importance of the clinic in providing support ranging from impersonal to supportive. “The clinic depends on the person. Some people want to go where they feel like a number, and some people want more warm and fuzzy” (Juliette). Liliana said that the chronic conditions her clients face often lead to perceived negative support from doctors, “them just saying very obnoxious things. I try to empower my clients to advocate for themselves.” Matteo agreed that his clients often reflected on the impersonal nature of medical support in infertility treatment, “they need a person, not a robot.” Overall, all participants agreed their clients have strong feelings about their clinics and the support they receive, with Juliette summarizing, “Unless they love their clinic, they’re not gonna stay, and unless they hate their clinic, they’re not gonna leave. There’s not a lot of room (for doubt), not as expensive, physical, and time consuming as it (infertiliy) is.”

The final type of support identified was financial support. Currently, insurance coverage for infertility is decided at the state level, with no federal mandate in place (RESOLVE, 2024). Liliana saw this as a significant barrier for her clients, saying, “We are the 50th state in terms of healthcare. There is no insurance coverage, and money dictates treatment.” Juliette and Maddi Lynn also spoke concerning the limitations of financial support dictating their client experience: “If I (Maddi Lynn) were going through this (infertility) now, I would go to Starbucks and get a job so I could have insurance.” This is especially true for Maddi Lynn’s same sex couples, who also have legal fees in addition to infertility treatment fees, impacting treatments like reciprocal IVF. Both participants from New England said financial support was more robust in their state, as a majority of New England states require insurance coverage for infertility. They both agreed that this relieved pressure from their clients to make medical decisions without worrying about the cost.

Within the imagery, support was minimally represented. Some participants included figures in their images, almost always a couple. For some participants, it was the combination of colors that represented the entire fertility process in a supportive way. Eloise noted all the colors in her image felt like the complete infertility experience, and “(couples and individuals) supporting each other.” Matteo also spoke about the mixture of colors in his image, in a supportive way, saying it felt like, “it is ten different colors, you know, so it is like ten different ideologies, providers, chiropractors, doing acupuncture, doing, doing, doing all that stuff.” Both Maddi Lynn and Matteo included a couple in their images as part of the infertility experience, while Juliette and Liliana did not include any representations of people. Matteo’s image included barriers and stated the puddle of blue at the bottom of the image “reflected like everything you’re throwing up the wall, to try all these certain things.” The puddle served as a mirror image that reflected “there are two sides to the coin.” His image moved out from the center, which reflects “your current present of all this chaos,” and at the end, he noted you “still have the couple, but they’re frustrated.” Eloise spoke about her image being the totality of the infertility experience, with the flowers representing the women she worked with in her groups and practice. She identified the movement she saw in her image as “a little bit of everything, like lots of different feelings all over the place…. Moments of joy in just connecting and not feeling alone.” No participant included family other than the couple, and there was no reference in the images of medical or financial support.

### 3.3 The long-term impact of infertility on individual and family mental health

The third central theme identified was the long-term impact of infertility on mental health, both for infertile individuals and their families. This impact was defined as the mental health concerns that continue to arise around baby-related activities and events, and the discussions that occur with nongenetic children as they grow into adulthood.

Participants talked about the overall long-term mental health impact of infertility, resulting from clients going through multiple treatment cycles or after a successful attempt at family building. Even clients who have had successful treatments note that “Nobody would choose to go down this path; it never leaves you” (Maddi Lynn). Matteo expressed the overall sentiment of the clients: ‘It is the Odyssey; it is just never-ending, and it is terrible. You keep going through these mythical adventures with no hope in sight. “Maddi Lynn also noted that the potential for more long-term impact on mental health has increased, now that critical infertility mental health services are being dropped from clinics when patients need the services the most. She stated, A lot of us (mental health clinicians) who were embedded are now not because private equity is buying out all these clinics, but because they don’t see we’re valuable; we’re the first to go.” Matteo and Maddi Lynn both spoke about the postpartum experience of mothers after infertility, particularly the approach to child safety and anxiety, such as sleeping safely or fear of losing the baby. Maddi Lynn identified “a higher incidence of (bad) postpartum when people have gone through infertility.”

Another long-term impact identified was the impact on children/the family. One participant in particular, Maddi Lynn, observed the impact infertility can have on children resulting from infertility treatments. With adoption and donor pregnancies playing large roles in infertility resolution, participants noted conversations are “not so black and white anymore” (Matteo). As the only participant currently working in clinics, Maddi Lynn said, “We’re just now understanding the needs of these children (donor conceived), and nobody pays attention to the children.” She has started to transition to more family support:

It’s really thinking about the needs of everybody: the donor, the gestational carrier, the future child, or the parent. I always say, “reach back out,” and now they are coming back when fertility is resolved, but it has not gone away.

Maddi Lynn hopes to pursue similar disclosure options to the adoption world, where genetic information and the adoption story are shared more openly. She identified new laws in two states she works with that champion open identity laws, “because nothing is secret anymore with ancestry.com. People have a genetic curiosity; it is actually good.” This is true for former successful infertility clients who never told their children they were donor-conceived. “They say I know I should have told my kids they were donor-conceived, but I didn’t know how. I always say it is better late than never. I think shame (for needing genetic donation) is definitely a part of that. (Maddi Lynn).”

When participants discussed their images, some of them included pieces that spoke to the resolution and long-term impact of infertility. Within the group of five participants, it was only the three therapists with a personal experience of infertility who drew about resolution and the future. Maddi Lynn notably spoke about what she had not included in her image, saying she “realized I didn’t really draw a baby anywhere. But then again, some people don’t have a baby in the end, you know?” She also talked about how the couple in her image started near the top of the page, while her image ended at the bottom, saying, “You don’t know where you’re gonna go.” Juliette’s image had a similar flow, also representative of the up-and-down nature of the infertility journey. Juliette also talked about the fragility of hope in her image, noting it was “overwhelming and it is buried, it is really buried amongst all those thorns in intensity, but it is still there.” She indicated her image started at the top of the page and moved down because “I always try to say to my clients, there will be health and happiness in your life.” Juliette noted it felt important to her to share that, because “you have a life after infertility.” Long-term impact was only represented for the core couple undergoing infertility, with no representation of the eventual family.

### 3.4 The personal impact of infertility on the clinicians

Three sub-categories emerged under clinician experience: the personal reproductive story in counseling, the sense of self in imagery, and treatment approaches currently used with infertility clients. Three of the participants had a personal history of infertility, while the others did not.

All clinicians spoke of their personal reproductive story in relation to their client’s experiences. The three participants who identified personal infertility all agreed it had an impact on their career choice, identifying it as a primary reason they chose to counsel infertility patients themselves. Maddi Lynn, who had experienced personal infertility, shared:

The beauty of infertility counseling, you know, is like it’s a club that no one ever thinks they are going to be in, and they do not want to join this club. But once you are in it, there are so many wonderful people and wonderful ones that have been before.

Eloise also experienced infertility, and during her personal counseling, she realized:

I need to do something about that because it’s just crazy that we are all going through this. We all know why we are in this room, yet no one can connect with each other. I wanted to help other people as they came through.

Regarding disclosure, participants chose to share personal experiences on a case-by-case basis. The three who had experienced personal infertility were more likely to disclose that fact, while the two without fertility issues shared only if asked. Those who did share felt it helped build trust and understanding with clients. Matteo noted he will “tell them I have kids if they ask me, but I don’t tell them I have kids. They’re here because they’re having problems; they don’t want to hear about my easy time.” Liliana noted the issue had never really come up, “they don’t really ask me. (about kids)” Juliette, who has a history of secondary infertility, shared that she discloses on a “case-by-case basis, because I see a lot of people with primary infertility, who don’t have kids yet.” Eloise and Maddi Lynn both said they disclose at the start of the work, with Eloise stating,

I feel it is important to disclose. I can relate to what they are going through and hold space for them. When I was going through it, I had a counselor disclose, and it made a difference for me to know they understood.

Eloise also noted that having a therapist who has an infertility history has been helpful for many of her clients, noting, “It can be exhausting to have to go through all the medical terms every time.” Maddi Lynn further shared in her experience, she “had a really bad counselor who didn’t understand and gave bad advice, like everything will be fine. But we were unexplained (no medical reason was found for infertility), so how did they know? So, I switched counselors.” Matteo and Liliana, neither with personal infertility, noted the experience of working with infertility clients changed how they approached fertility in general. Matteo said he “is now more aware of how and what I ask, I don’t ask when people are having kids now.” Liliana shared she “hasn’t thought about my own journey yet. Still, I know the other side of it now, and I think about it.”

As participants talked about their images, the three participants with infertility noted a sense of self in the image they created, with all three noting it felt unconscious. Within the imagery, pieces of their own experiences were interwoven into their images, mostly through their color choices. Maddi Lynn used orange frequently throughout her image, noting “orange is my daughter’s favorite color and that is where my story started. It is hard to take myself out of it (the image)”. Juliette, another clinician with an infertility past identified she “could see myself in this image- once you have been there it is hard to not think you know how it all goes.” Her image had green as a hopeful color, with hope being something “I leaned on and encourage my clients too as well.” The final participant with an infertility past, Eloise, identified similar ideas through symbolism in her use of flowers. Her comment was delivered almost as an afterthought, that she saw herself “as one of the flowers in the field”. No participant noted reflection of their experience overtaking the client experience, with Eloise noting “if I didn’t know my own story, I wouldn’t see it in there, but because I do, I am mixed in.” Both clinicians without an infertility history made no mention of seeing themselves in their final images, only identifying the client experience. Participants who did see themselves in the image noted a mix of symbols and colors, primarily using colors that had emotional meaning in their experience.When asked about treatment, the treatment approaches used by the five clinicians varied. Two participants were expressive therapists who used expressive approaches with their clients. The remaining three used a variety of mental health approaches, such as cognitive behavioral therapy (CBT), bibliotherapy, and mindfulness for infertility-related issues.

Cognitive-based therapy was the most common approach used by therapists, being one of the standard treatments for mental health issues. Juliette and Matteo both noted there are some challenges with CBT- “There’s a decent amount of let’s do the work in CBT to get what you want. And so that is a delicate balance, because this isn’t necessarily going to lead to a pregnancy.” Matteo said his clients felt CBT “could be cold…they need a human to talk to about this.” In addition to CBT, Juliette uses “some mindfulness, some positive psychology, some Adlerian sprinkled in there. Our past doesn’t have to define us. It influences us, but we don’t have to say this is who I am and bummer.” Maddi Lynn and Matteo both said they focused mainly on being a place to vent and on helping direct people to services when needed. Maddi Lynn said it was “a little bit like the old social worker stuff (in arranging resources and referrals),” and Matteo said he “just sees himself as a place they can come to vent and be angry.”

Eloise and Liliana both identified primarily as expressive therapists and mainly used expressive interventions with clients. Eloise runs workshops with a friend who does infertility yoga, and they “incorporate guided imagery and art therapy into it.” She talked about encouraging mindfulness, and “just checking in with yourself to really see what you need.” Liliana, a registered art therapist, said much of her work with clients is art-based. She said she “will sometimes just pick a song, or the client will pick a song if the moment feels right. We do art with that.” Liliana talked about an intervention she calls rage art: “We smash things; we experiment with different textures. We have done a lot of art around body parts.” Liliana was the only participant who identified using music in her work, alongside the art. Eloise also discussed using art in her work, saying, “They (the clients) bring in photos, work they have done in between sessions. Kind of intense images of despair and grief.”

While not trained as expressive therapists, Matteo, Juliette, and Maddi Lynn did identify some expressive techniques, such as journaling, guided imagery, and drawing in sessions. They all noted these treatments helped shift perspective, with Matteo stating, “No one’s cookie-cutter, I can’t do something cookie-cutter, you have to kind of base it on the person.” Juliette discussed a journaling intervention she has clients do, exploring what they have lost in infertility. She shared she “has had clients come back and tell me it was hard, it was painful, but no one has ever come back and told me it was a waste of time.”

### 3.5 Reflection of client experience through music and imagery

The final theme identified was Reflection of Client Experience with two small, but distinct subthemes: facilitation of the imagery through the music and possible use with clients. All five participants were asked about their artistic experience and how the use of music with the imagery assisted in facilitating the image. Multiple participants identified the music as something that put them in the right mindset and pull them out of a cognitive space. Maddi Lynn noted, “It was a lot in the beginning…. I am one of those people who go kicking and screaming to get out of my head. And the reality is that’s what the music did (got me out of my head).” Eloise noted her clients have used images before, and the music “forces you to slow down a little bit, give (clients) a chance to step out of that thinking space.” Eloise further noted the music “felt like I could be present and relaxed, which isn’t always the case.” The form and shape of the music was discussed by multiple participants, identifying numerous points in the music that helped them engage more fully in the imagery part of the experience. Matteo spoke about his experience in the music shifting his image and perspective, sharing, “at first, I wasn’t focused on the music, when I was about halfway through the first part of the picture, there was a shift in the music. And it brought up the not so positive side into the image.” The three participants without an expressive arts background found the music to be particularly helpful in decreasing nervousness or fear about drawing, allowing them to fully reflect on the experiences of their clients.

When asked about the possible benefits an arts-based method such as music and imagery might provide to clients, Liliana and Eloise both noted imagery can help a client express emotion that is harder to verbalize, with Liliana saying, “my clients have verbalized that being able to express those emotions has been helpful for them…. Having an outlet or being able to show their doctors like how they’re feeling as opposed to just telling them.” Eloise concurred, “I do think it could be helpful, just to kind of, you know, describe how they’re feeling, maybe when it is harder to do in words.” Juliette also agreed using music and imagery could be helpful for her clients, saying, “(Music and imagery) could be helpful, yes, very much so… (the music) allowed for a calm overall that allowed me to… it was an asset to the outlet (imagery).” Matteo concurred saying it could be an interesting experience for his clients, though not all. Maddi Lynn said she thought it could be very beneficial, noting she has used art therapy previously with children. She said she had not thought of using the technique with infertility patients, saying, “I haven’t thought about using it with this population, but I should.”

## 4 Discussion

This study asked the following questions: (1) What are the experiences of infertility clinicians reflecting on their clients’ infertility experiences, and (2) How did the use of artistic engagement shape the reflection process? Results indicate a mixture of experiences from infertility clients: loss, support, long-term impact, and the clinician’s personal experience concerning fertility. Examining the experience of infertility through artistic engagement via music and imagery, results indicated that the addition of music and imagery aided in identifying their clients’ infertility struggles, particularly through colors and symbolism commonly used by those experiencing infertility. Clinicians were chosen as participants in this study to assess the music and imagery technique prior to the introduction of this technique to the wider infertility community, and to use the artistic methods to assist clinicians in easing the emotional burden of infertility, something identified in previous literature (Fancourt et al., 2020).

### 4.1 Experience of loss

Participants identified loss as a common experience brought to counseling. This is consistent with previous studies on the mental health impact of infertility, with research on stigma and identity tied to the reproductive process having indicated that infertility causes extreme mental stress (Kucukkaya and Kilic, 2022; Öztürk et al., 2021; Szatmári et al., 2021). Participants noted their clients’ losses came in many forms: emotional loss, loss of children (or the hope of children), loss of social support, loss of trust in their bodies, and loss of confidence in medical situations. The loss of children/loss of hope of children through the malfunction of the body is something particularly present in current infertility literature, with research focused on both the inability to conceive and carry as well as recurrent miscarriages. Some studies focused on the specifics of marginalized communities’ experiences, such as Ceballo et al. (2015), which examined the experience of Black women, while others were more general (Crockett et al., 2021; Galeotti et al., 2022; Gao et al., 2020). Recurrent miscarriage has been defined in previous studies as the inability to carry multiple pregnancies, which impacts how women perceive themselves as women, mothers, and wives (Bailey et al., 2019; Linehan et al., 2023; Pillarisetty and Mahdy, 2023). This challenge to self-identity creates a lasting mental health impact that can be difficult to overcome. Emotional loss, or the grief that comes from being unable to conceive or carry children, is another common experience of infertility, one often ignored or disenfranchised. Studies on grief, specific to infertility, have shown couples and individuals experiencing infertility feel isolated from grief communities and expected to manage their grief in solitude (James and Singh, 2018; McBain and Reeves, 2019; Tian and Solomon, 2020).

Many of the identified losses are well documented in current literature, and this study reinforces this knowledge in two important areas: the long term impact loss can have, as well as the impact infertility and pregnancy loss can have on social relationships socially when faced with infertility and pregnancy loss. In addition, this study adds to the current literature through the exploration and discussion around complicated loss and disenfranchised loss, topics that are currently underserved in reproductive research. While studies have started to explore the impact disenfranchised and silent grief can have on mental health, the discussion of clinicians serving this population is something not currently explored in research. The clinician voice in this regard is important, as it identifies issues facing the community through clear examples, and allows for a clinical approach to treating and addressing disenfranchised reproductive grief.

Imagery completed in a music and imagery exercise was consistent with the verbal identifications of loss, taking up a large portion of all participant images. This data corroborates loss as an encompassing theme in infertility, spreading across all areas of daily and long-term life. While there have been previous studies using relaxation and guided imagery methods (Aulakh et al., 2024; Damghanian et al., 2025; Mustafa et al., 2021; Park and Kim, 2026), this study continues to provide more data in the concurrent use of arts-based methods along more traditional interviews to gain more understanding of the infertility experience across multiple modes.

### 4.2 Support

Participants identified a lack of social support as the driving desire to avoid painful reminders of pregnancy or children at social events. Study participants all identified their clients’ struggle to attend baby showers or interact with friends who were pregnant or mothers while they were experiencing pregnancy loss or were unable to conceive. Numerous studies document the struggles faced in social situations of those going through infertility, including loss of friends and family, and struggles at work. Abraham (2019) interviewed participants from infertility support groups to identify behaviors that were identified as helpful or not helpful for those experiencing infertility. Borowczak and Rotoli (2022) identified that men and women had different primary concerns in support: men said they felt overlooked and stigmatized, while women felt isolated, stigmatized, and were the recipients of unhelpful comments. All participants discussed various aspects of support, with the consensus being that their clients experienced high levels of stigmatization and isolation. Matteo, the only clinician routinely seeing male clients, corroborated the experience of his clients, which was the same as those identified in Borowczak and Rotoli: the central theme being stigma and inability to seek support for themselves over their partner. This lack of social comfort and support can create a devastating impact on individuals going through infertility, causing them to isolate and not seek the support of trusted friends and family.

When discussing the types of support experienced by clients undergoing infertility treatment, participants separated support by categories: male versus female and individual versus group or couple. Just as the process of infertility is markedly different between men and women (Cosson et al., 2022; Lucia and David, 2022; Hanna and Gough, 2020; Holley et al., 2015; Karavolos, 2016), so is the support process. While most participants worked with women, one participant also worked with men who were diagnosed with male factor infertility. He noted that, in his experience, men were less concerned with their own emotional or mental processing and more focused on their partner. He also noted male clients in his practice were not comfortable talking about their own emotions and found the issue of fertility to be emasculating. Participants’ views in the study correlate with other studies focused on male infertility: men see infertility as emasculating and primarily a woman’s issue (Cervi and Knights, 2022; Garcia, 2021; Harlow et al., 2020).

For female clients, participants identified common mental aspects of infertility, such as supporting emotions, stress, anxiety, and depression (Anaman-Torgbor et al., 2021; Dube et al., 2021; Holley et al., 2015; Taebi et al., 2021; Yokota et al., 2022). Treatment methods used by the participants with their infertility patients were in line with those used generally to treat anxiety and depression: cognitive behavioral therapy (CBT) and mindfulness (Dube et al., 2021; Faramarzi et al., 2013; Galhardo et al., 2019; Golshani et al., 2021; Kalhori et al., 2020). For individual support, participants focused on each person’s experience of emotion and grief in the client’s story. In contrast, participants identified group support as focused more on the process of lifting each other’s spirits and offering support.

Additionally, participants discussed the role of financial support during treatment and how finances affect the overall trajectory of options for individuals with infertility. The cost of *in vitro* fertilization (IVF) is prohibitive, and the lack of insurance coverage for the procedure often limits access to personalized infertility treatment (Fantus, 2021; Fantus and Newman, 2022; Klitzman, 2017; Su, 2022). For example, participants noted how finances limited treatment options for their clients, including clients in need of genetic donations. While many of the results confirmed current and past literature, clinician discussion around the male experience, and more particularly, the financial impact are important points, as both these topics are under reported in current research.

The imagery created by participants did not reference support outright, but many included representations of a couple on the infertility path. This data offers a differing view of how clinicians may see support, versus how the clients may feel supported. When reflecting on client experiences in interview, participants noted clients did not often mention their partners in session, focusing mostly on their own symptoms. Participants verbally identified that their male and female clients had very different needs, and it was primarily the female or carrying partner that presented for treatment. If the couple came together, participants confirmed the focus of the treatment remained on the woman or carrying partner. However, the imagery included by some participants highlighted the couple, suggesting clinicians may not fully see the isolation a client may be feeling. More study in this area would provide more understanding of the complex role of support in the infertility process.

### 4.3 Long-term impact

The overall long-term impact of infertility on both mental health and parenting is noted in recent literature as long-lasting and impactful, even when treatments may have been successful or other options have been utilized to address infertility (Bell, 2019; Chauhan et al., 2020). There is a lack of therapeutic support in general for couples and women who remain childless, with a lack of clinicians trained in the long-term impacts of infertility a significant concern (Fieldsend and Smith, 2020; Gold, 2013). In addition to the long-term effects on clients, one participant noted the emerging impact on children created through non-genetic means, such as donor material or surrogacy. Results from this study added to the current literature through the discussion around the long-term impact of infertility on children, particularly around the disclosure of genetic origins to children. This is a conversation that has grown recently in the world of adoption (Van Den Akker, 2001; Bell, 2019; Drustrup, 2016; Janus, 1997) but is still growing in regard to infertility resolution through other means such as surrogacy or genetic donation as identified by participants in this study.

Imagery in this area was included only by the participants with a personal infertility history, suggesting the long-term impact of their own experience may influence the support they offer and counsel their clients on. Imagery around the long-term impact mostly focused on the negative outcome of treatment, as no participant included a baby or family in their image. One participant specifically noted that you do not always have a child, and another mentioned she tries to have her clients focus on the future with or without a child. Notably, no participant reflected on the long-term impact to the family in their image, despite one participant verbally identifying such a shift occurring in her practice. This suggests clients are not returning to counseling to address the long-term impacts of infertility on either themselves or their family, despite studies identifying a variety of issues well after active fertility treatment has ended. The two participants without a personal infertility history only reflected on client experiences in the present, suggesting they may not be as aware of the long-term impacts and therefore focus more on the feelings present in active treatment. Further study in this area would be useful in both exploring the mental health experience of families created via genetic donation or adoption, as well as developing education for infertility counselors to better understand the long-term impacts on both the individual and the family.

### 4.4 Impact on clinicians

The fourth finding was the impact on the clinicians who work with infertility clients. Most of the study’s participants experienced some form of infertility themselves and cited this experience as a primary reason for choosing their field of work. This acknowledgement reflects the influence of the personal reproductive story (Jaffe, 2017), and participants who did not experience personal infertility noted the impact this work has had on their perception of fertility in others. Jaffe (2017) pointed out that the role of personal reproductive stories influences counseling procedures, and participants in this study demonstrated this in terms of disclosure and approach to counseling. The treatment methods used by the participants also reflected their approach to infertility. Although using tested methods like CBT and mindfulness, the clinicians realized these methods may feel cold and unfeeling to their clients. Wallis (2021) found this to be the case in clinicians they interviewed as well, while more unorthodox methods, expressive therapies, and positive psychology were effective for specific clients. Overall, participants noted that a combination of approaches was most effective, a finding endorsed by the Dube et al. (2021) study. A notable finding in the clinician experience was identified in the imagery, though only with the participants with a personal infertility history. All three participants in this category all saw some sense of themselves in the images created around their clients’ experiences, saying it was hard to separate their stories from the work they do. All participants felt this was not a conscious choice, but more a result of their infertility being so closely tied to their choice to counsel this population. Future study on the impact of personal history on infertility counselors would offer more insight into the ability to separate personal history and reduce bias.

### 4.5 Artistic experience of infertility

Using artistic engagement to describe the infertility experience has not been explored in depth. Due to limited research on using expressive treatment methods for infertility, there is limited data on the role of artistic engagement in infertility treatment. While previous studies using art and psychodrama techniques have been published (Hughes and Da Silva, 2011; Streeter and Deaver, 2018; Terzioğlu and Özkan, 2018), this study is among the first to use music and imagery specifically for treating infertility. To explore the use of this treatment technique, clinicians were asked to complete a music and-imagery experience on their clients’ infertility experiences. When asked about the artistic experience, participants identified music as an aid in defining the client’s infertility experience and helping the participants express their clients’ infertility experience artistically and authentically.  Music and imagery was not used as music therapy intervention in this study, nor was it used as BMGIM or CMGIM intervention. Through the exploratory use of music and imagery as an arts-based method, it may be that a music therapy or guided imagery protocol could be developed for use with the infertility population.

### 4.6 Music

Participants noted that the musical piece felt like a mirror of the infertility experience and that the art created held significant meaning within the infertility community. Study participants noted important symbols and colors and used these in art to express ideas and concerns facing their clients. While there is no literature supporting the use of music and imagery in infertility treatment, the identified experience from the participants in this study mirrors that of reported results when using music and imagery in other traumatic situations requiring counseling, such as working with refugees and veterans of war (Beck et al., 2018; Story and Beck, 2017). Music and imagery have also been used in the past with anxiety and depression, symptoms which individuals with infertility frequently experience (Beck et al., 2015; Ip-Winfield and Grocke, 2021).

### 4.7 Color and symbolism

The use of color and symbolism is an essential aspect of imagery and can serve as a container for feelings and emotions that may otherwise be too hard to discuss (Beebe and Wyatt, 2009; Chen, 2024; Küssner and Eerola, 2019; Summer, 2010). The participants in this study gravitated toward specific imagery and colors, indicating that color is associated with their (and their clients’) personal infertility experiences and emotions. This finding suggests experience specific imagery and color is of reproductive mental health processing. Previous studies in music and imagery for mental health treatment indicate that using music and art aids in the processing and release of trauma and emotions (Beck et al., 2018; Montgomery, 2012; Story and Beck, 2017; Summer, 2010).

#### 4.7.1 Participant art

Participants in this study used the art to identify the experience of infertility as shared with them by their clients (Supplementary Appendix 3). In their artwork, participants often shared similar imagery and colors that they identified as related to the infertility experience. Despite an exhaustive search, there was no literature in which to compare the participant’s artwork and meanings. Participants in this study drew a variety of barrier representations, such as mountains and valleys. All participants in this study indicated their clients often talked about barriers with their bodies, their relationships, and their medical treatments. In the two attached pictures, there are clear boundaries and obstacles to achieving the goal of pregnancy, a constant theme in the participants’ work. All participants also included some image or symbol to represent the client or couple. Maddi Lynn used hearts to represent her clients, while Matteo used mirror images of his clients. Liliana used birds to represent her clients, and Juliette drew people. These symbols often carried double meanings, with hearts symbolizing both the person and the emotional significance of a pregnancy. Liliana noted her birds were symbols both for clients and hope.

In addition to the symbolism, colors were an essential part of the artistic experience for the participants. The participants gave the colors emotional importance and often served a dual purpose, representing both the infertility experience and the emotions involved. All participants used red in some way, red meaning both loss from blood and love. Some participants noted green as a color with dual meanings: envy and earth and grounding. Many participants used pinks and blues, noting that clients felt hopeful about the most preferred outcome of infertility: a baby boy or girl. Black was used in many of the participant images as well, most often representing a barrier that cannot be overcome or deep grief. All the participants in this study included colors and symbols that were closely intertwined, with the symbol representing a part of the journey and the color representing the emotional process of the infertility journey.

### 4.8 Contribution to current music therapy and GIM research

The results of this study use music and imagery as the artistic method of exploration and could potentially be a foundation for developing a reproductive trauma specific music therapy or guided imagery protocol. The use of music and imagery in this study expands the use of music-based activities more into the realm of reproductive mental health. While there have been recent studies using music therapy to address reproductive health during infertility and attempts to get pregnant (Short et al., 2025), there is no research that uses music therapy or guided music and imagery to address the mental health and emotional needs of reproductive trauma. With further development, it is possible the use of music and imagery could be developed into a music therapy intervention that could be used in reproductive mental health. Studies have demonstrated the ability of expressive arts to allay some symptoms of reproductive trauma (Hughes and Da Silva, 2011; Streeter and Deaver, 2018), and past music therapy research has demonstrated parallels to addressing symptoms of mental health in reproductive trauma such as depression, anxiety, grief, and post-traumatic stress disorder (Bar et al., 2023; Erkkilä et al., 2021; Finnerty et al., 2023). Additionally, recent studies have explored the use of relaxation (Aulakh et al., 2024; Damghanian et al., 2025; Mustafa et al., 2021; Park and Kim, 2026). The combination of music and imagery in this study expands reproductive mental health in the field of expressive treatments for infertility, and potentially offers a baseline for further development into more specific clinical applications. Additionally, while current research on the Bonney Method of Guided Music and Imagery and the Continuum Method of Guided Music and Imagery have explored the use of these techniques in mental health and along parallel lines to reproductive trauma (Beck et al., 2018a,b; Beck et al., 2015), this is the first study to explore elements of this technique specifically with reproductive trauma and mental health. Further research would be beneficial to continue building knowledge on the impact and use of music and imagery to possibly create music therapy and guided imagery techniques for use in reproductive trauma and health.

### 4.9 Limitations

This study faced several limitations that should be considered in future studies. One significant limitation was the lack of diversity among the sample population. Infertility is an individual journey that shares similarities across certain aspects, such as treatment. Currently, infertility counseling is primarily conducted by individuals identifying as White, which skews the views and potentially discounts the experiences of marginalized communities. In the future, expanding the sampling pool is needed to include greater diversity and include communities beyond the White infertility experience.

Additionally, the sample population in this study was gender-biased, consisting mainly of women. Historically, men have been underserved in infertility research (Harlow et al., 2020). Future studies that include the experiences of male clinicians and the treatment and support of men diagnosed as are is needed to bring more understanding of the differences between the male and female infertility experience.

To explore the use of expressive therapies with infertility clients, it was determined to first attain the views and experiences of infertility clinicians on the use of these therapies to assess the safety and emotional risk. However, if the use of music and imagery in infertility treatment is not conducted with individuals currently seeking infertility counseling, the approach is isolated and limited in its research value. While conducting this research with clinicians may not be a limitation, it does rely on the client’s secondhand experience of infertility as reported by the clinician. Future studies should include participants actively in infertility treatments, independently or in a group, to verify the information being provided by mental health clinicians.

### 4.10 Recommendations

There is currently not enough literature discussing the needs of non-traditional infertility cases, such as LGBTQIA or single individuals who fall into both categories of social and medical infertility, to determine if their emotional, social, mental, and financial needs are adequately supported. Further study on the long-term impacts of infertility would be beneficial, as there are indicators that infertility is never truly resolved, even following the cessation of treatments. In future studies, asking participants why they select specific symbols or colors will help provide insight into why images and colors are essential for creating imagery and whether these elements contribute to the release of strong emotions.  Research with individuals using participant-selected music would also provide another layer of insight into the personal infertility experience.

## 5 Conclusion

Infertility is a global problem that affects both men and women, physically and mentally. Individuals experiencing infertility seek treatment for different types of loss, varying levels of support, and long-term impacts on mental health. Working with individuals experiencing infertility, clinicians’ views on fertility were personally impacted, whether they were personally experiencing infertility or not. Current treatment for the mental aspect of infertility is based on the standard methods of cognitive behavioral therapy and mindfulness. However, studies show a variety of treatment methods work best (Dube et al., 2021). There is currently limited research regarding artistic and expressive methods for treating individuals with infertility.

Results from this study indicate a profound impact on participants using expressive techniques, demonstrating the potential of adding these methods to infertility treatments. While this study had limitations that need to be addressed in future research, notably by expanding the sample’s diversity, results indicated that music and imagery helped describe and provide a view of the infertility experience for these participants. The use of artistic methods including music and imagery in addressing the mental health aspects of infertility is an underexplored area, and this study offered valuable insights into its role. As such, these participants identified music and imagery as a potential strategy in addressing infertility distress symptoms: anxiety, depression, and trauma. Future research focusing on the use of music and imagery with individuals experiencing infertility will be a valuable addition to the arsenal of current methods treating infertility distress.

## Data Availability

The raw data supporting the conclusions of this article will be made available by the authors, without undue reservation.
