# Supplementary material for: Music, imagery, and infertility: a qualitative inquiry into symptoms, treatments, and expressive therapies with infertility clinicians
Source: Front Psychol. 2026 Jun 23;17:1778519. doi: 10.3389/fpsyg.2026.1778519 (PMC13337694; doi:10.3389/fpsyg.2026.1778519)
Supplement: Supplementary file 1 [file Supplementary_file_1.DOCX]

**Appendix A**

**Prompt**

“Please close your eyes or soften your gaze and take a few deep breaths in and out. I will now play a piece called The Hudson by Johannes Martens 4 times through. It will be repeated and simply start again when it concludes. As the music is playing, I invite you to reflect on the experiences of your clients in their infertility work and create any imagery that comes to mind.”

**Music**

The Hudson, Johannes Martens. Performed by Ola Gjeilo
