# Supplementary material for: Music, imagery, and infertility: a qualitative inquiry into symptoms, treatments, and expressive therapies with infertility clinicians
Source: Front Psychol. 2026 Jun 23;17:1778519. doi: 10.3389/fpsyg.2026.1778519 (PMC13337694; doi:10.3389/fpsyg.2026.1778519)
Supplement: Supplementary file 2 [file Supplementary_file_2.DOCX]

Appendix B

**Interview Questions**

- Not all questions may be asked

Prompt: I am now going to ask you some questions about your infertility work and clients in general terms, to identify trends in current symptoms and presentations in infertility mental health. I will then ask some questions about the music and imagery experience you just completed, to gather data on your experience. Some of the questions may feel very personal, so please let me know if you do not feel you can answer any question, and we will move on.

Demographic Questions

1. What region/state do you practice in?
2. If you feel comfortable, what gender do you identify with?
3. If you feel comfortable, what race do you identify with?
4. How many years have you been in practice?
   1. How many with infertility clients in particular?
5. What licensure do you have?
   1. What is your highest level of education?
6. Have you personally experienced infertility as a patient?
   1. If yes, can you briefly describe your experience?
   2. If yes, did you seek counseling throughout your infertility experience?
   3. If yes, did you resolve your infertility?
   4. If yes, did this impact your choice to counsel infertility patients?
7. If you did not experience infertility personally, why did you choose to work with infertility clients?

Infertility Questions

1. Have you ever heard the term infertility distress?
   1. If yes, can you tell me where and how you have heard it used?
   2. If yes, can you tell me your personal definition of infertility distress?
   3. If no, how would you define infertility distress
2. Please describe your current practice
   1. Are there symptoms you see more than others?
   2. Do you see both men and women?
   3. If yes, can you describe some of the differences in approach?
3. Is recurrent miscarriage a symptom you often see in your practice?
4. Do you specialize in one area of infertility? For example, LGBTQIA, donor egg, primary or secondary infertility?
5. What treatment techniques do you use most with your clients?
   1. What have you found to be successful about these techniques?
   2. Have you noted any challenges with these techniques?
6. Have you ever used expressive or creative therapy approaches with your clients?
7. Have you ever worked, or do you work with couples?
   1. If yes, can you describe some of the differences between 1:1 sessions and couples?
   2. If yes, have you noticed symptoms of distress that are unique to couples?
8. Have you ever run group sessions or support groups?
   1. If yes, can you describe in general terms some of the differences in group sessions versus 1:1 or dyad sessions?
9. Have you noticed a difference in presentation or engagement in treatment in regards to culture?
10. Have you noticed a difference in presentation or engagement in treatment in regards to race?
11. If you have experienced personal infertility, can you share how that has impacted your approach to counseling?
12. Is there anything you would like to add, or want others to know about the infertility experience?

Music and Imagery Questions

1. Have you engaged in creative or expressive interventions such as this one before?
   1. If yes, can you please describe it?
2. Can you tell me how the music and imagery experience felt for you?
3. Have you ever engaged in a prompted music and imagery experience before?
4. While you were listening, did you find it hard to create drawn imagery?
   1. If yes, do you have a sense of why?
   2. If no, can you identify what felt easy about drawing to the music?
5. How did you find yourself reflecting on the infertility experience while you were listening and drawing?
6. Do you feel the music or imagery impacted your reflections in any way?
   1. If yes, can you tell me how?
   2. If no, was there a reason you felt it did not?
   3. If yes, was there a particular symptom or client that you found yourself reflecting on? In general terms, if it was a client, was there something about their story that you found yourself reflecting on?
7. Did you notice any emotions or feelings emerge as you were listening and drawing?
8. If you have experienced personal infertility, did the music and imagery bring up reflections of your personal experience?
   1. If yes, do you feel comfortable sharing what you were reflecting on?
9. I would like to ask about the style of the music. Can you tell me how you experienced the style and instrumentation? For example, did you notice anything in your body, or in the artwork you created?
10. After participating in this music and imagery experience, do you feel expressive or creative interventions would be useful in working with infertility clients?
11. Is there anything else you would like to add about the music and imagery experience?
12. Is there anything I haven’t asked that you would like to add?

Thank you so much for your time and willingness to share. Please let me know if I can assist you in any way or if you need additional support.
