# Supplementary figures and images for: Music, imagery, and infertility: a qualitative inquiry into symptoms, treatments, and expressive therapies with infertility clinicians
Source: Front Psychol. 2026 Jun 23;17:1778519. doi: 10.3389/fpsyg.2026.1778519 (PMC13337694; doi:10.3389/fpsyg.2026.1778519)

**
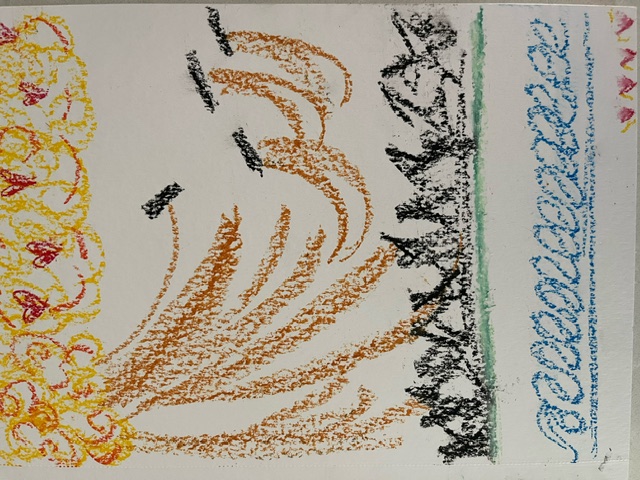
**Two of the images completed by participants

**
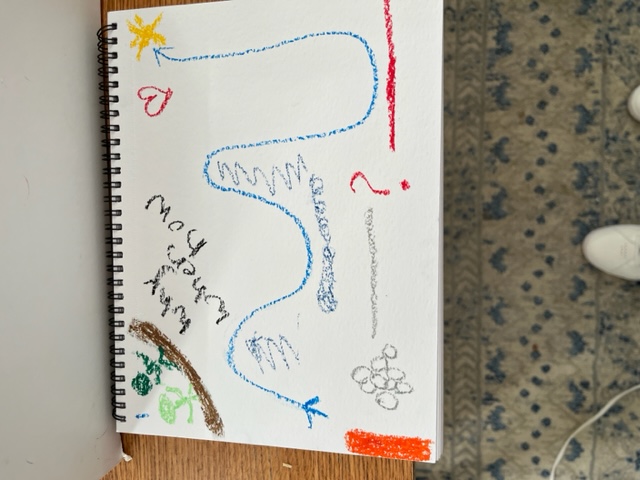
**

Supplement: Supplementary file 3 [file Supplementary_file_3.docx]
